# A second monoclinic polymorph of *N*-cyclo­hexyl-*N*-ethyl­benzene­sulfonamide

**DOI:** 10.1107/S160053680904762X

**Published:** 2009-11-18

**Authors:** Zeeshan Haider, Muhammad Nadeem Arshad, Muhammad Zia-ur-Rehman, Islam Ullah Khan, Muhammad Shafiq

**Affiliations:** a Department of Chemistry, Government College University, Lahore 54000, Pakistan; b Applied Chemistry Research Centre, PCSIR Laboratories Complex, Ferozpure Road, Lahore 54600, Pakistan

## Abstract

The crystal structure of the title compound, C_14_H_21_NO_2_S, is a polymorph of the structure reported by Khan *et al.* [*Acta Cryst.* (2009), E**65**, o2867] which is also monoclinic (space group *P*2_1_/*c*). The unit cell in the title structure is approximately half the volume of the previously reported polymorph and the asymmetric unit of the title compound contains one mol­ecule rather than two independent mol­ecules in the other polymorph. In the title mol­ecule, the cyclo­hexane ring is in the typical chair form. In the crystal structure, mol­ecules are linked *via* weak inter­molecular C—H⋯O inter­actions, forming a chain along the *b*-axis direction.

## Related literature

For the synthesis of related mol­ecules, see: Arshad *et al.* (2009 ▶); Zia-ur-Rehman *et al.* (2009 ▶). For applications of sulfonamides, see: Connor (1998 ▶); Berredjem *et al.* (2000 ▶); Lee & Lee (2002 ▶); Xiao & Timberlake (2000 ▶). For the structure of the other polymorph, see: Khan *et al.* (2009 ▶). For standard bond-length data, see: Allen *et al.* (1987 ▶).
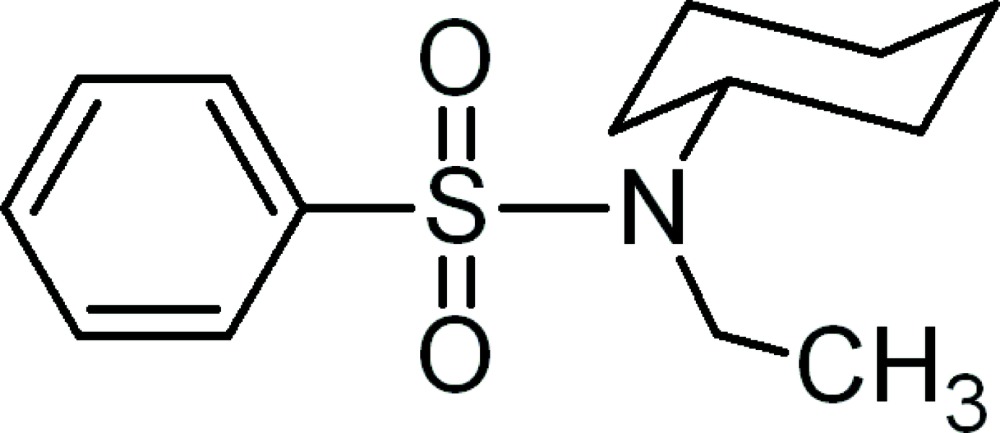


## Experimental

### 

#### Crystal data

C_14_H_21_NO_2_S
*M* *_r_* = 267.38
Monoclinic, 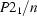

*a* = 8.3837 (4) Å
*b* = 11.4467 (5) Å
*c* = 15.1488 (7) Å
β = 92.541 (2)°
*V* = 1452.34 (12) Å^3^
*Z* = 4
Mo *K*α radiation
μ = 0.22 mm^−1^
*T* = 296 K
0.41 × 0.28 × 0.11 mm

#### Data collection

Bruker APEXII CCD diffractometer
Absorption correction: multi-scan (*SADABS*; Sheldrick, 2007 ▶) *T*_min_ = 0.916, *T*_max_ = 0.976
15910 measured reflections
3651 independent reflections
2481 reflections with *I* > 2σ(*I*)
*R*_int_ = 0.034

#### Refinement

*R*[*F* ^2^ > 2σ(*F* ^2^)] = 0.042
*wR*(*F* ^2^) = 0.107
*S* = 1.02
3651 reflections
164 parameters
H-atom parameters constrained
Δρ_max_ = 0.26 e Å^−3^
Δρ_min_ = −0.36 e Å^−3^

### 

Data collection: *APEX2* (Bruker, 2007 ▶); cell refinement: *SAINT* (Bruker, 2007 ▶); data reduction: *SAINT*; program(s) used to solve structure: *SHELXS97* (Sheldrick, 2008 ▶); program(s) used to refine structure: *SHELXL97* (Sheldrick, 2008 ▶); molecular graphics: *PLATON* (Spek, 2009 ▶) and *Mercury* (Macrae *et al.*, 2006 ▶); software used to prepare material for publication: *WinGX* (Farrugia,1999 ▶) and *PLATON*.

## Supplementary Material

Crystal structure: contains datablocks I, global. DOI: 10.1107/S160053680904762X/lh2950sup1.cif

Structure factors: contains datablocks I. DOI: 10.1107/S160053680904762X/lh2950Isup2.hkl

Additional supplementary materials:  crystallographic information; 3D view; checkCIF report

## Figures and Tables

**Table 1:** Hydrogen-bond geometry (Å, °)

*D*—H⋯*A*	*D*—H	H⋯*A*	*D*⋯*A*	*D*—H⋯*A*
C6—H6⋯O2^i^	0.93	2.58	3.482 (2)	163

Symmetry code: (i) 
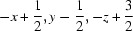
.

**Fig. 1.**
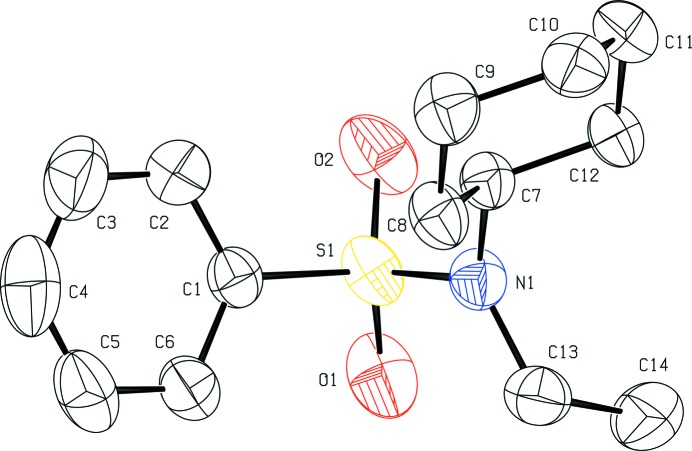
The molecular structure of (I), with displacement ellipsoids at the 50% probability level.

**Fig. 2.**
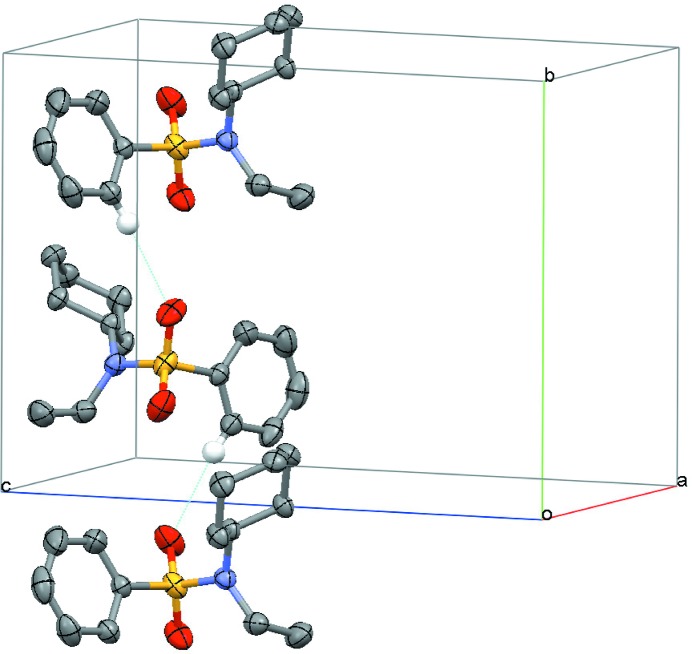
Part of the crystal structure showing weak C-H···O hydrogen bonds as dashed lines. H atoms not involved in hydrogen bonding have been omitted for clarity.

## supplementary crystallographic information

### Comment

Sulfonamides are an important category of pharmaceutical compounds with a broad spectrum of biological activities such as herbicidal, anti-malarial, anti-convulsant and anti-hypertensive (Connor, 1998; Xiao & Timberlake, 2000; Berredjem *et al.*, 2000; Lee & Lee, 2002).

As a part of our ongoing research program regarding the synthesis of sulfur containing heterocyclic compounds (Arshad *et al.*, 2009; Zia-ur-Rehman *et al.* 2009), we, herein report the crystal structure of the title compound as a new polymorph of the structure previously reported by Khan *et al.* (2009) which is also monoclinic (space group *P*2_1_/*c*), but with completely different unit cell constants. The molecular structure of the title compound (I) is shown in Fig. 1. The asymmetric unit contains single molecule instead of two as observed in the previous form. In the molecule of (I), bond lengths (Allen *et al.*, 1987) and bond angles are within the normal ranges. The cyclohexane ring is in the chair form. In the crystal structure, molecule are linked via weak intermolecular C—H···O hydrogen bonds to form chains along the b axis direction.

### Experimental

A mixture of *N*-cyclohexylbenzene sulfonamide (1.0 g, 0.43 mmol), sodium hydride (0.21 g; 0.88 mmoles) and *N*, *N*-dimethylformamide (10.0 ml) was stirred at room temperature for half an hour followed by addition of ethyl iodide (0.134 g; 0.86 mmoles). Stirring was continued further for a period of three hours and the contents were poured over crushed ice. Precipitated product was isolated, washed and crystallized from methanol.

### Refinement

All hydrogen atoms were identified in a difference Fourier map. However, they were fixed in ideal positions and treated as riding on their parent atoms. The following distances were used: C_methyl = _—H 0.98 Å. C_aromatic_—H 0.95 Å. U(H) was set to 1.2U_eq_(C) or 1.5U_eq_(C_methyl_).

### Crystal data

**Table:**

C_14_H_21_NO_2_S	*F*(000) = 576
*M**_r_* = 267.38	*D*_x_ = 1.223 Mg m^−^^3^
Monoclinic, *P*2_1_/*n*	Mo *K*α radiation, λ = 0.71073 Å
Hall symbol: -P 2yn	Cell parameters from 4610 reflections
*a* = 8.3837 (4) Å	θ = 2.8–27.6°
*b* = 11.4467 (5) Å	µ = 0.22 mm^−^^1^
*c* = 15.1488 (7) Å	*T* = 296 K
β = 92.541 (2)°	Needle, colourless
*V* = 1452.34 (12) Å^3^	0.41 × 0.28 × 0.11 mm
*Z* = 4	

### Data collection

**Table:**

Bruker APEXII CCD diffractometer	3651 independent reflections
Radiation source: fine-focus sealed tube	2481 reflections with *I* > 2σ(*I*)
graphite	*R*_int_ = 0.034
φ and ω scans	θ_max_ = 28.5°, θ_min_ = 2.2°
Absorption correction: multi-scan (*SADABS*; Sheldrick, 2007)	*h* = −11→10
*T*_min_ = 0.916, *T*_max_ = 0.976	*k* = −15→14
15910 measured reflections	*l* = −19→20

### Refinement

**Table:**

Refinement on *F*^2^	Primary atom site location: structure-invariant direct methods
Least-squares matrix: full	Secondary atom site location: difference Fourier map
*R*[*F*^2^ > 2σ(*F*^2^)] = 0.042	Hydrogen site location: inferred from neighbouring sites
*wR*(*F*^2^) = 0.107	H-atom parameters constrained
*S* = 1.02	*w* = 1/[σ^2^(*F*_o_^2^) + (0.0409*P*)^2^ + 0.3342*P*] where *P* = (*F*_o_^2^ + 2*F*_c_^2^)/3
3651 reflections	(Δ/σ)_max_ = 0.001
164 parameters	Δρ_max_ = 0.26 e Å^−^^3^
0 restraints	Δρ_min_ = −0.36 e Å^−^^3^

### Special details

**Table:**

Geometry. All e.s.d.'s (except the e.s.d. in the dihedral angle between two l.s. planes) are estimated using the full covariance matrix. The cell e.s.d.'s are taken into account individually in the estimation of e.s.d.'s in distances, angles and torsion angles; correlations between e.s.d.'s in cell parameters are only used when they are defined by crystal symmetry. An approximate (isotropic) treatment of cell e.s.d.'s is used for estimating e.s.d.'s involving l.s. planes.
Refinement. Refinement of *F*^2^ against ALL reflections. The weighted *R*-factor *wR* and goodness of fit *S* are based on *F*^2^, conventional *R*-factors *R* are based on *F*, with *F* set to zero for negative *F*^2^. The threshold expression of *F*^2^ > σ(*F*^2^) is used only for calculating *R*-factors(gt) *etc*. and is not relevant to the choice of reflections for refinement. *R*-factors based on *F*^2^ are statistically about twice as large as those based on *F*, and *R*- factors based on ALL data will be even larger.

### Fractional atomic coordinates and isotropic or equivalent isotropic displacement parameters (Å^2^)

**Table:**

	*x*	*y*	*z*	*U*_iso_*/*U*_eq_	
S1	0.22296 (5)	0.78592 (4)	0.73395 (3)	0.04988 (15)	
O1	0.14310 (16)	0.68075 (13)	0.70635 (9)	0.0702 (4)	
O2	0.14198 (15)	0.89484 (12)	0.72376 (10)	0.0720 (4)	
N1	0.38527 (16)	0.79318 (12)	0.68134 (9)	0.0455 (3)	
C1	0.27998 (18)	0.77256 (14)	0.84693 (10)	0.0427 (4)	
C2	0.2929 (3)	0.87099 (18)	0.89914 (13)	0.0658 (5)	
H2	0.2641	0.9436	0.8760	0.079*	
C3	0.3483 (3)	0.8612 (2)	0.98515 (15)	0.0841 (7)	
H3	0.3569	0.9276	1.0205	0.101*	
C4	0.3908 (3)	0.7554 (3)	1.01939 (14)	0.0809 (7)	
H4	0.4296	0.7497	1.0777	0.097*	
C5	0.3767 (2)	0.6566 (2)	0.96771 (14)	0.0710 (6)	
H5	0.4048	0.5843	0.9916	0.085*	
C6	0.3209 (2)	0.66398 (16)	0.88051 (12)	0.0528 (4)	
H6	0.3113	0.5975	0.8454	0.063*	
C7	0.49240 (18)	0.89465 (14)	0.69898 (10)	0.0410 (4)	
H7	0.4309	0.9530	0.7304	0.049*	
C8	0.6390 (2)	0.86509 (15)	0.75812 (11)	0.0507 (4)	
H8A	0.6055	0.8330	0.8136	0.061*	
H8B	0.7026	0.8065	0.7296	0.061*	
C9	0.7397 (2)	0.97408 (17)	0.77604 (11)	0.0579 (5)	
H9A	0.6786	1.0303	0.8085	0.069*	
H9B	0.8340	0.9536	0.8121	0.069*	
C10	0.7892 (2)	1.02824 (16)	0.69050 (12)	0.0547 (5)	
H10A	0.8578	0.9744	0.6605	0.066*	
H10B	0.8495	1.0990	0.7033	0.066*	
C11	0.6449 (2)	1.05710 (15)	0.63070 (12)	0.0550 (4)	
H11A	0.6803	1.0871	0.5750	0.066*	
H11B	0.5828	1.1177	0.6580	0.066*	
C12	0.53986 (19)	0.95047 (14)	0.61323 (10)	0.0457 (4)	
H12A	0.4446	0.9735	0.5789	0.055*	
H12B	0.5971	0.8939	0.5790	0.055*	
C13	0.4471 (2)	0.68754 (15)	0.63861 (12)	0.0542 (5)	
H13A	0.4181	0.6195	0.6726	0.065*	
H13B	0.5627	0.6916	0.6396	0.065*	
C14	0.3849 (3)	0.67214 (18)	0.54449 (13)	0.0760 (6)	
H14A	0.2704	0.6677	0.5429	0.114*	
H14B	0.4273	0.6014	0.5208	0.114*	
H14C	0.4172	0.7374	0.5098	0.114*	

### Atomic displacement parameters (Å^2^)

**Table:**

	*U*^11^	*U*^22^	*U*^33^	*U*^12^	*U*^13^	*U*^23^
S1	0.0385 (2)	0.0606 (3)	0.0500 (3)	−0.0046 (2)	−0.00434 (17)	0.0147 (2)
O1	0.0615 (8)	0.0877 (10)	0.0603 (8)	−0.0332 (7)	−0.0110 (6)	0.0087 (7)
O2	0.0469 (7)	0.0815 (10)	0.0880 (10)	0.0182 (7)	0.0050 (7)	0.0353 (8)
N1	0.0459 (8)	0.0483 (8)	0.0421 (7)	−0.0072 (6)	0.0015 (6)	0.0003 (6)
C1	0.0378 (8)	0.0476 (9)	0.0431 (9)	−0.0015 (7)	0.0050 (7)	0.0043 (7)
C2	0.0839 (14)	0.0548 (12)	0.0599 (12)	0.0012 (10)	0.0160 (10)	−0.0002 (9)
C3	0.1083 (19)	0.0908 (18)	0.0541 (13)	−0.0248 (15)	0.0138 (12)	−0.0155 (12)
C4	0.0689 (14)	0.131 (2)	0.0427 (11)	−0.0152 (14)	−0.0031 (10)	0.0049 (13)
C5	0.0665 (13)	0.0872 (16)	0.0594 (12)	0.0133 (12)	0.0045 (10)	0.0285 (12)
C6	0.0543 (10)	0.0529 (11)	0.0513 (10)	0.0005 (9)	0.0064 (8)	0.0090 (8)
C7	0.0408 (8)	0.0433 (9)	0.0387 (8)	−0.0020 (7)	0.0010 (6)	0.0009 (7)
C8	0.0509 (10)	0.0581 (11)	0.0422 (9)	−0.0051 (8)	−0.0066 (7)	0.0079 (8)
C9	0.0556 (11)	0.0678 (13)	0.0494 (10)	−0.0100 (9)	−0.0087 (8)	−0.0030 (9)
C10	0.0516 (10)	0.0528 (11)	0.0599 (11)	−0.0123 (8)	0.0033 (8)	−0.0052 (8)
C11	0.0592 (11)	0.0480 (10)	0.0580 (11)	−0.0046 (8)	0.0052 (9)	0.0101 (8)
C12	0.0456 (9)	0.0512 (10)	0.0399 (9)	−0.0009 (8)	−0.0014 (7)	0.0087 (7)
C13	0.0606 (11)	0.0449 (10)	0.0567 (11)	−0.0022 (8)	−0.0024 (9)	0.0017 (8)
C14	0.1067 (18)	0.0615 (13)	0.0591 (12)	−0.0003 (12)	−0.0042 (12)	−0.0115 (10)

### Geometric parameters (Å, °)

**Table:**

S1—O2	1.4247 (13)	C8—C9	1.524 (2)
S1—O1	1.4309 (14)	C8—H8A	0.9700
S1—N1	1.6093 (14)	C8—H8B	0.9700
S1—C1	1.7632 (16)	C9—C10	1.511 (2)
N1—C13	1.476 (2)	C9—H9A	0.9700
N1—C7	1.485 (2)	C9—H9B	0.9700
C1—C2	1.378 (2)	C10—C11	1.515 (2)
C1—C6	1.381 (2)	C10—H10A	0.9700
C2—C3	1.368 (3)	C10—H10B	0.9700
C2—H2	0.9300	C11—C12	1.521 (2)
C3—C4	1.359 (3)	C11—H11A	0.9700
C3—H3	0.9300	C11—H11B	0.9700
C4—C5	1.377 (3)	C12—H12A	0.9700
C4—H4	0.9300	C12—H12B	0.9700
C5—C6	1.384 (3)	C13—C14	1.507 (3)
C5—H5	0.9300	C13—H13A	0.9700
C6—H6	0.9300	C13—H13B	0.9700
C7—C12	1.516 (2)	C14—H14A	0.9600
C7—C8	1.527 (2)	C14—H14B	0.9600
C7—H7	0.9800	C14—H14C	0.9600
			
O2—S1—O1	119.34 (9)	H8A—C8—H8B	108.1
O2—S1—N1	108.08 (8)	C10—C9—C8	110.74 (14)
O1—S1—N1	107.08 (8)	C10—C9—H9A	109.5
O2—S1—C1	106.77 (9)	C8—C9—H9A	109.5
O1—S1—C1	108.29 (8)	C10—C9—H9B	109.5
N1—S1—C1	106.64 (7)	C8—C9—H9B	109.5
C13—N1—C7	119.89 (13)	H9A—C9—H9B	108.1
C13—N1—S1	119.71 (11)	C9—C10—C11	111.09 (15)
C7—N1—S1	117.98 (11)	C9—C10—H10A	109.4
C2—C1—C6	120.85 (16)	C11—C10—H10A	109.4
C2—C1—S1	119.81 (14)	C9—C10—H10B	109.4
C6—C1—S1	119.21 (13)	C11—C10—H10B	109.4
C3—C2—C1	119.6 (2)	H10A—C10—H10B	108.0
C3—C2—H2	120.2	C10—C11—C12	111.69 (14)
C1—C2—H2	120.2	C10—C11—H11A	109.3
C4—C3—C2	120.6 (2)	C12—C11—H11A	109.3
C4—C3—H3	119.7	C10—C11—H11B	109.3
C2—C3—H3	119.7	C12—C11—H11B	109.3
C3—C4—C5	120.01 (19)	H11A—C11—H11B	107.9
C3—C4—H4	120.0	C7—C12—C11	111.15 (13)
C5—C4—H4	120.0	C7—C12—H12A	109.4
C4—C5—C6	120.6 (2)	C11—C12—H12A	109.4
C4—C5—H5	119.7	C7—C12—H12B	109.4
C6—C5—H5	119.7	C11—C12—H12B	109.4
C1—C6—C5	118.32 (18)	H12A—C12—H12B	108.0
C1—C6—H6	120.8	N1—C13—C14	113.41 (15)
C5—C6—H6	120.8	N1—C13—H13A	108.9
N1—C7—C12	110.79 (12)	C14—C13—H13A	108.9
N1—C7—C8	113.40 (13)	N1—C13—H13B	108.9
C12—C7—C8	111.16 (13)	C14—C13—H13B	108.9
N1—C7—H7	107.0	H13A—C13—H13B	107.7
C12—C7—H7	107.0	C13—C14—H14A	109.5
C8—C7—H7	107.0	C13—C14—H14B	109.5
C9—C8—C7	110.38 (14)	H14A—C14—H14B	109.5
C9—C8—H8A	109.6	C13—C14—H14C	109.5
C7—C8—H8A	109.6	H14A—C14—H14C	109.5
C9—C8—H8B	109.6	H14B—C14—H14C	109.5
C7—C8—H8B	109.6		
			
O2—S1—N1—C13	−146.72 (13)	C2—C1—C6—C5	−0.6 (3)
O1—S1—N1—C13	−16.96 (14)	S1—C1—C6—C5	175.29 (14)
C1—S1—N1—C13	98.80 (13)	C4—C5—C6—C1	−0.1 (3)
O2—S1—N1—C7	50.94 (13)	C13—N1—C7—C12	66.92 (17)
O1—S1—N1—C7	−179.30 (11)	S1—N1—C7—C12	−130.77 (12)
C1—S1—N1—C7	−63.54 (13)	C13—N1—C7—C8	−58.89 (19)
O2—S1—C1—C2	−21.71 (17)	S1—N1—C7—C8	103.43 (15)
O1—S1—C1—C2	−151.40 (15)	N1—C7—C8—C9	−177.88 (13)
N1—S1—C1—C2	93.66 (16)	C12—C7—C8—C9	56.52 (19)
O2—S1—C1—C6	162.38 (13)	C7—C8—C9—C10	−57.4 (2)
O1—S1—C1—C6	32.69 (16)	C8—C9—C10—C11	56.9 (2)
N1—S1—C1—C6	−82.25 (15)	C9—C10—C11—C12	−55.4 (2)
C6—C1—C2—C3	0.6 (3)	N1—C7—C12—C11	178.00 (13)
S1—C1—C2—C3	−175.27 (16)	C8—C7—C12—C11	−54.96 (19)
C1—C2—C3—C4	0.1 (3)	C10—C11—C12—C7	54.4 (2)
C2—C3—C4—C5	−0.7 (4)	C7—N1—C13—C14	−107.93 (18)
C3—C4—C5—C6	0.7 (3)	S1—N1—C13—C14	90.06 (18)

### Hydrogen-bond geometry (Å, °)

**Table:**

*D*—H···*A*	*D*—H	H···*A*	*D*···*A*	*D*—H···*A*
C6—H6···O2^i^	0.93	2.58	3.482 (2)	163

Symmetry codes: (i) −*x*+1/2, *y*−1/2, −*z*+3/2.
